# Evolutionary Insights into IL17A in Lagomorphs

**DOI:** 10.1155/2015/367670

**Published:** 2015-12-15

**Authors:** Fabiana Neves, Joana Abrantes, Tereza Almeida, Paulo P. Costa, Pedro J. Esteves

**Affiliations:** ^1^CIBIO, InBIO-Research Network in Biodiversity and Evolutionary Biology, Universidade do Porto, Campus de Vairão, Rua Padre Armando Quintas, 4485-661 Vairão, Portugal; ^2^Unidade Multidisciplinar de Investigação Biomédica (UMIB), Universidade do Porto (UP), Rua de Jorge Viterbo Ferreira, No. 228, 4050-313 Porto, Portugal; ^3^Departamento de Genética, CSPGF, Instituto Nacional de Saúde Dr. Ricardo Jorge, Rua Alexandre Herculano, No. 321, 4000-055 Porto, Portugal; ^4^Departamento de Biologia, Faculdade de Ciências da Universidade do Porto, Rua do Campo Alegre, s/n, 4169-007 Porto, Portugal; ^5^Centro de Investigação em Tecnologias de Saúde (CITS), CESPU, Rua Central de Gandra, No. 1317, 4585-116 Gandra, Portugal

## Abstract

In leporids, IL17A had been implicated in the host defense against extracellular pathogens, such as* Francisella tularensis* that infects hares and rabbits and causes the zoonotic disease tularemia. Here, we studied IL17A from five lagomorphs, European rabbit, pygmy rabbit, brush rabbit, European brown hare, and American pika. We observed that this protein is highly conserved between these species, with a similarity of 97–99% in leporids and ~88% between leporids and American pika. The exon/intron structure, N-glycosylation sites, and cysteine residues are conserved between lagomorphs. However, at codon 88, one of the interaction sites between IL17A and its receptor IL17RA, there is an Arg>Pro mutation that only occurs in European rabbit and European brown hare. This could induce critical alterations in the IL17A structure and conformation and consequently modify its function. The differences observed between leporids and humans or rodents might also represent important alterations in protein structure and function. In addition, as for other interleukins, IL17A sequences of human and European rabbit are more closely related than the sequences of human and mouse or European rabbit and mouse. This study gives further support to the hypothesis that European rabbit might be a more suitable animal model for studies on human IL17.

## 1. Introduction

Interleukin 17, first known as cytotoxic T lymphocyte associated antigen (CTLA) 8, is originated from a T-cell derived factor with cytokine-like activity [1, 2]. With a ubiquitous expression in different tissues, this protein, nowadays known as IL17A, has a sequence composition different from all the other cytokine families [1, 3]. IL17A, along with five functional homodimers (IL17B-F), one heterodimer (IL17A/F), and 5 receptors (IL17RA-RE), composes the IL17 family, which is important to adaptive immunity responses, namely, as mediator of chronic inflammation and autoimmune diseases [3–6]. There is a wide range of genes that are targeted by IL17, such as proinflammatory and hematopoietic cytokines, genes associated with acute phase response, and antimicrobial substances [3, 7]. This protein is also part of a subset of CD4 T helper (Th) cells known as Th17 which are able to establish a connection between innate and adaptive immune responses, being a complement to Th1 and Th2 defense mechanisms [8]. Furthermore, the production of IL17A is important for host defense against extracellular pathogens (fungi, viruses, bacteria, and parasites) assisting in neutrophils recruitment and activation and also promoting antimicrobial peptides [8–12]. Studies in mice [12–15] and humans [16–18] highlighted the importance of IL17 expressing cells for immunity against several diseases, and low expression levels of IL17 and IL17RA make organisms more susceptible to disease, including those caused by extracellular pathogens such as* Francisella tularensis.*

*F. tularensis* is highly pathogenic Gram negative intracellular bacteria included by the Center of Disease Control and Prevention (CDC) in category A of bioterrorism (http://emergency.cdc.gov/agent/agentlist.asp). Able to cause the zoonotic disease tularemia, this microorganism has several known hosts, from mammals to protozoans; however transmission to humans is normally associated with direct contact with lagomorphs, rodents, and some arthropods [15, 20–22]. In lagomorphs and rodents,* F. tularensis* has the ability to cause septicemia while in humans the outcome of infection is a multisystem organ failure [23]. There are several reports of* F. tularensis* infections in leporids, mainly in rabbits (European rabbit and cottontails) [24–26] and hares [24, 26, 27] and despite an apparent period of stasis (2006–2010) there were some recently documented outbreaks of tularemia in Europe [22, 28].

The order Lagomorpha includes two families, Leporidae (rabbits and hares) with eleven genera and Ochotonidae (pikas) with only one genus,* Ochotona* [29]. Together with rodents, lagomorphs form the clade Glires, a sister group of Euarchonta that includes primates [30, 31]. Along with mouse, the European rabbit had been used as a research model for several human diseases, development of therapeutics and vaccines [32]. Several studies have suggested that the European rabbit may be a better research model than mouse [33–37]. With the exception of humans and mouse, there is a big gap of information on IL17A in other mammalian groups, including leporids. Thus, considering the important biological role of the European rabbit immune response against several diseases, including tularemia, we performed a genetic characterization of IL17A in four leporid genera (*Oryctolagus*,* Brachylagus*,* Sylvilagus*, and* Lepus*).

## 2. Material and Methods

**Figure 1:**
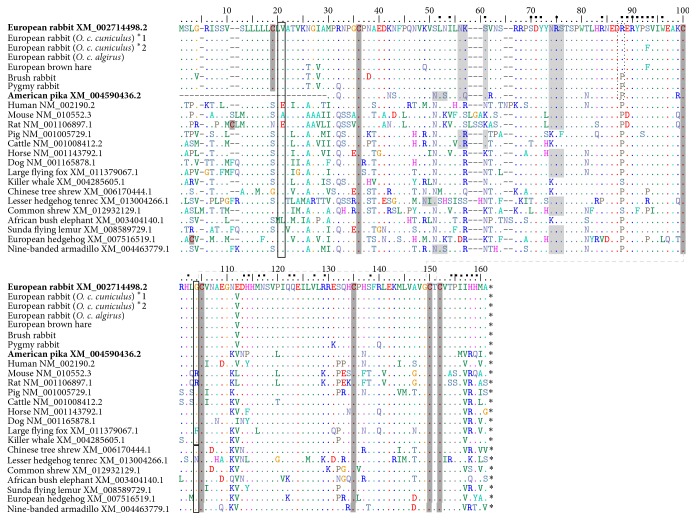
Alignment of IL17A for several mammalian species. GenBank and Ensembl accession numbers are indicated in bold for the retrieved sequences. Positively selected amino acids are boxed (according to [19]). N-glycosylation sites are shaded in light grey and cysteine residues are shaded in dark grey. A black dashed box represents the Agr>Pro mutation between leporids. *∗* represents stop codons; − represents indels; ▪ above the numbering represents the sites important for IL17A-IL17Ra interaction. ^*∗*^1 and ^*∗*^2 represent different alleles. Numbering is according to the European rabbit sequence (GenBank accession number XM_002714498.2) and the signal peptide and indels were included in the numbering. Disulfide bonds between side chain cysteines are represented by a light grey dashed line.

Samples of European rabbit (*Oryctolagus cuniculus cuniculus and Oryctolagus cuniculus algirus*), pygmy rabbit (*Brachylagus idahoensis*), brush rabbit (*Sylvilagus bachmani*), and European brown hare (*Lepus europaeus*) were provided by the CIBIO Lagomorpha tissue collection. Genomic DNA (gDNA) was extracted using the EasySpin Genomic DNA Minipreps Tissue Kit (Citomed, Torun, Poland) according to the manufacturer's instructions. Total RNA was extracted by using the RNeasy Mini Kit also according to the manufacturer's instructions (Qiagen, Hilden, Germany) from one specimen of European rabbit and one of European brown hares. Complementary DNA (cDNA) was synthesized using oligo(dT) as primers and SuperScript III reverse transcriptase (Invitrogen, Carlsbad, CA, USA). The European rabbit and American pika IL17A sequences were retrieved from public databases (accession numbers are given in bold in Figure 1). PCR amplification was performed with the Multiplex PCR Kit (Qiagen) by using two pairs of primers designed according to the retrieved sequences (for genomic DNA F1-CGTCCAACCTCAGTTGATC + R1-CACTGTACCATCTATCCTGC and F2-CCTTCATTTACTCCCATTCG + R2-CATCCATCACATGGCCTAA; for cDNA the combination of primers F1 + R2 was used). Sequencing was performed on an ABI PRISM 310 Genetic Analyzer (PE Applied Biosystems, Foster City, CA, USA) and PCR products were sequenced in both directions. The sequences obtained were submitted to GenBank with the following accession numbers: KU163611–KU163619.

Haplotype phases of the sequences obtained were reconstructed with the program PHASE, built into the software DnaSP [38]. Multiple Sequence Comparison by Log-Expectation (MUSCLE; http://www.ebi.ac.uk/) [39] was used for sequence alignment. The putative N-glycosylation sites were predicted using NetNGlyc 1.0 (http://www.cbs.dtu.dk/services/NetNGlyc/) [40].

The number of nucleotide differences per site between sequences was estimated in MEGA6 [41] with the following options: bootstrap method (1000 replicates), p-distance as model, and pairwise deletion for gaps/missing data treatment. A Maximum Likelihood approach was used to estimate the phylogenetic relationships between the IL17A nucleotide sequences by using MEGA6; the best-fit nucleotide substitution model was predicted by the same software and 1000 bootstrap replicates were used.

The secondary structure of IL17A was predicted using PsiPred (http://bioinf.cs.ucl.ac.uk/psipred/) [42, 43] and DiAminoacid Neural Network Application (DiANNA) (http://clavius.bc.edu/~clotelab/DiANNA/) [44]. Both methods predict protein cysteines that create disulfide bonds, but while PsiPred uses Position Specific Iterated-BLAST (PSI-BLAST) to obtain evolutionary information used to predict the secondary structure of the query protein, DiANNA is a neural network that recognizes cysteines in an oxidized state (sulfur covalently bonded) distinguishing them from those in a reduced state.

## 3. Results and Discussion

In this study we amplified and sequenced the IL17A gene for four leporids species (European rabbit, European brown hare, brush rabbit, and pygmy rabbit). For European rabbit (*O. c. cuniculus*) and European brown hare, both genomic and cDNA sequences were identical and only one of the sequences is presented; however both sequences have been assigned different accession numbers. These sequences were further compared to sequences of IL17A from another lagomorph, American pika (*Ochotona princeps*), and from representatives of the most relevant mammalian groups (e.g., Artiodactyla, carnivores, Chiroptera, Primates, rodents, etc.) available in online databases. In the European rabbit, IL17A is located in the forward strand of chromosome 12 and has a similar structure to other mammals with three coding exons. The IL17A cDNA sequence obtained in this work for* Lepus europaeus* showed a similar structure.

**Figure 2:**
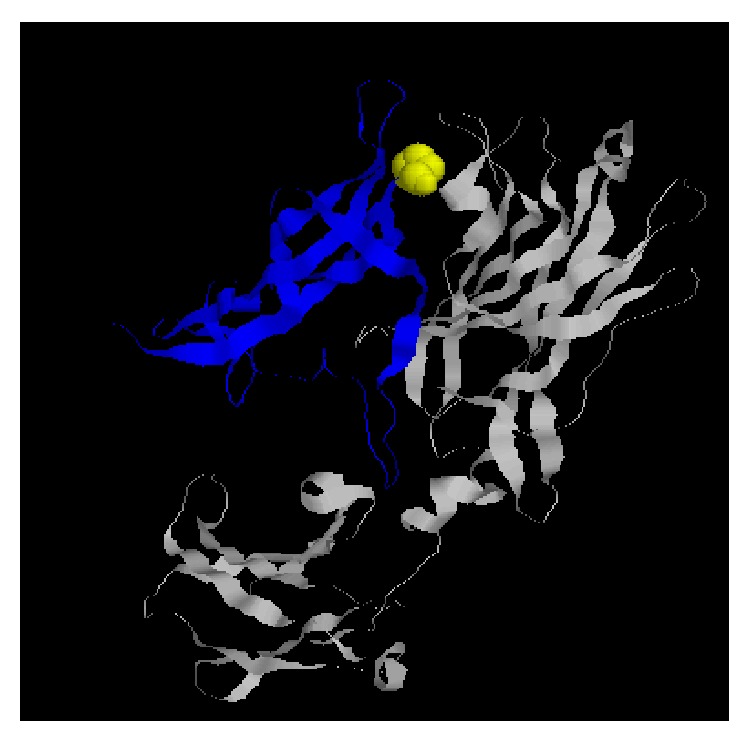
3D structures of the IL17A-IL17RA complex. IL17A appears in blue while IL17RA appears in grey. Marked in yellow is the 88Arg>Pro mutation described for leporids.

In humans, IL17A codes for a protein with 155 amino acids (aa) and has the ability to bind with high affinity to IL17RA and IL17RC [6, 45, 46]. The interaction between interleukins and their receptors is crucial for their function and signaling and any changes in the amino acid composition may induce alterations in the protein conformation. In humans and rodents these interactions sites are described [6, 45] and include Leu52, Ile54, Ser61, Ser70-Tyr72, Arg75, Arg84, Arg88-Val94, Trp96, Leu103, His114, His115, Asn117, Ser118, Gln122-Glu124, Leu128, Arg130, Phe139, and Pro155-Met160 (Figure 1). In leporids, IL17A codes for a protein with 153 aa and we observed that the sites that likely interact with the receptors are quite conserved. Indeed, from the thirty-three amino acids involved in the linkage between IL17A and IL17RA, eighteen are conserved: twelve are maintained between mammals and the other six, despite being different, do not alter the charge or the polarity. For the remaining fifteen amino acids, only three are differently charged, seven have distinct polarity, and five have both different charge and polarity (Table 1). Between leporids these sites are highly conserved, but a mutation was observed that is located in the external coil of the IL17A in a site where this protein interacts with IL17RA (Figure 2). This mutation, 88Arg>Pro, occurs in the European rabbit and in the European brown hare, while in brush rabbit and the American pika the amino acid present is a proline as in most mammals. Some studies showed that Arg>Pro mutations have crucial effects in the protein function [47–49]. Indeed, the 332Arg>Pro mutation in human Trim5*α* restricts infection by HIV-1 (Human Immunodeficiency Virus-1) [49] while the 132Arg>Pro mutation in the helicase protein of coronavirus infectious bronchitis virus was lethal to infectivity* in vitro* [48]. Additionally, this mutation alters the physiochemical properties of the amino acid by changing from a basic polar and positively charged arginine to a nonpolar and neutral proline.

Disulfide bounds and N-glycosylation sites (Asn-X-Ser/Thr/Cys motifs where X can be any amino acid except proline) are important for the protein structure, stability, and function [50–52]. Disulphide bounds occur between cysteines side chains and these linkages are also important for protein protection [53]. In human and rodents, IL17A has a cysteine knot fold characterized by two sets of paired *β*-strands (1/2 and 3/4) interconnected by two disulfide bounds between strands 2 and 4 linked between four conserved cysteines (Cys100–Cys150 and Cys105–Cys152) [45, 54, 55]. In addition to these cysteines two other cysteines are common to all mammals, Cys36 and Cys135. For the European rabbit, the PsiPred predicted secondary structure and the DiANNA predicted disulfide bonds results are in agreement with those obtained and described for human and rodents [45, 54]. An extra linkage is also predicted between Cys31 and Cys135. When compared to other mammals, there is an extra cysteine (Cys19) in leporids located in the signal peptide. The rat and the European hedgehog also have an extra cysteine located in different sites of the signal peptide (Cys11 and Cys3, resp.). Given that the signal peptide is cleaved in order for the protein to become active, this extra cysteine should not have an impact on the IL17A structure.

N-glycosylation is a crucial factor for the modulation of protein activity; therefore, alteration on these sites may interfere with recognition of targets, including receptors, and consequently affects the biological activity of the proteins and also their ability to diffuse through the organism [56, 57]. Human IL17A is N-glycosylated at Asn68. Detection of putative N-glycosylation sites indicated that this N-glycosylation site is present in the majority of mammals, including rodents and lagomorphs. Other putative N-glycosylation sites were detected and include Asn56 in lagomorphs, pig, and cattle, Asn51 in American pika and armadillo, and Asn49 in the lesser hedgehog tenrec. The killer whale and the African bush elephant have no putative N-glycosylation sites. The implications of the absence/presence of N-glycosylation sites in IL17A are unknown; however some studies indicate that presence/removal of glycans in some proteins do not alter their folding or function, although a decrease in the protein dynamics is observed [50, 52, 57].

**Figure 3:**
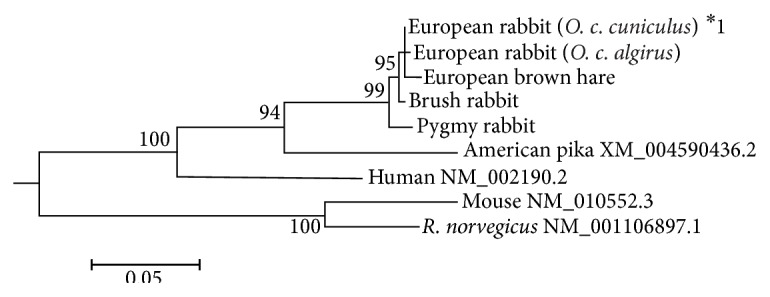
Maximum Likelihood (ML) tree of the IL17A nucleotide sequences. Only bootstrap values ≥ 94% are shown. In order to facilitate visualization, only one sequence/allele of each species was used.

Comparison of the nucleotide sequences (Table 2) indicated that, in leporids, the European rabbit and the European brown hare IL17A sequences are the least divergent (0.011) while the European rabbit and the pygmy rabbit IL17 sequences are the most divergent (0.026). Between the European rabbit and American pika, the genetic diversity obtained was 0.112–0.115. For the remaining mammals the highest divergence occurs for the lesser hedgehog tenrec (0.312) and the lowest divergence for the flying lemur (0.145). The comparison of the nucleotide diversity of several interleukins in the European rabbit suggested that it could represent a better animal model for research [34]. For IL17A, similar results were obtained, with the human sequence being more closely related to the European rabbit (0.169) than to mouse or rat IL17A sequences (0.236). This is further supported by a Maximum Likelihood tree inferred for IL17A mammalian sequences (Figure 3).

## 4. Conclusions

In the present study we sequenced and characterized IL17A for four leporids. Overall, the genomic organization, the location of the cysteine residues, and the presence of N-glycosylation sites are highly conserved in leporids. Nevertheless, a single mutation was detected within the interaction site with IL17RA which may induce crucial changes in IL17A structure, function, stability, signaling, and conformation. Further functional and structural studies should be performed to fully understand the impact of this specific mutation. The lowest divergence between the European rabbit and human IL17A sequences reinforces the hypothesis that the European rabbit might be a more suitable animal model for studies in the human innate immunity.

## Figures and Tables

**Table 1:** Characterization of the IL17A amino acids differences in the sites important for binding to IL17RA.

Amino acid position	Amino acids				
	Leporids				Other mammals
	European rabbit	European brown hare	Brush rabbit	Pygmy rabbit	
52	L ^#^				M^#^, S^*∗*^
54	I ^#^				V^#^, S^*∗*^, T^*∗*^
61	S^*∗*^				N ^*∗*^, K^*∗*+^
70	S ^*∗*^				T^*∗*^, L^#^
71	D^*∗*−^				
72	Y^*∗*^				
75	R^*∗*+^				
84	R ^*∗*+^				P^#^, V^#^
88	R^*∗*+^		P^#^		P ^#^, S^*∗*^
89	E ^*∗*−^				D^*∗*−^
90	R^*∗*+^				
91	Y ^*∗*^				F^#^
92	P ^#^				S^*∗*^
93	S ^*∗*^, F^#^	F^#^	S^*∗*^		F^#^, P^#^, R^*∗*+^
94	V^#^				
96	W ^#^				L^#^
103	L ^#^				Q^*∗*^, S^*∗*^, M^#^
114	H^*∗*+^				P^#^, Y ^*∗*^, F^#^, L^#^
115	H^*∗*+^				
117	N^*∗*^				
118	S^*∗*^				
122	Q ^*∗*^				K^*∗*+^
123	Q^*∗*^				
124	E^*∗*−^				
128	L^#^				
130	R ^*∗*+^				K^*∗*+^
139	F^#^				
155	P ^#^				S^*∗*^
156	I ^#^				M^#^
157	I^#^				V ^#^
158	H ^*∗*+^				S^*∗*^, R^*∗*+^, Q^*∗*^, K^*∗*+^
159	H ^*∗*+^				Q^*∗*^, Y^*∗*^, T^*∗*^
160	M^#^				I^#^, V ^#^, A, L^#^

The amino acid polarity (^*∗*^hydrophilic; ^#^hydrophobic) and charge (^+^positive; ^−^negative) are properly annotated. The amino acid present in the human IL17A sequence is underlined. Numbering is according to the European rabbit IL17A sequence.

**Table 2:** IL17A nucleotide distances (the lowest values are in bold and the highest values are underlined).

	1	2	3	4	5	6	7	8	9
(1) European_rabbit (*O. c. cuniculus*)	—								
(2) European_rabbit (*O. c. algirus*)	0.002	—							
(3) European brown hare	**0.011**	0.013	—						
(4) Brush rabbit	**0.011**	0.013	0.013	—					
(5) Pygmy rabbit	0.024	0.026	0.026	0.022	—				
(6) American pika XM_004590436.2	0.112	0.115	0.120	0.112	0.112	—			
(7) Human NM_002190.2	**0.169**	0.171	0.175	0.171	0.173	0.159	—		
(8) Mouse NM_010552.3	0.251	0.251	0.249	0.249	0.245	0.240	0.236	—	
(9) Rat NM_001106897.1	0.260	0.260	0.258	0.258	0.253	0.232	0.236	0.111	—
